# Fine-Needle Aspiration Cytology of Soft Tissue Sarcoma: Benefits and Limitations

**DOI:** 10.1080/13577149877911

**Published:** 1998-12

**Authors:** Måns Åkerman

**Affiliations:** Department of Pathology and Cytology University Hospital Lund S-221 85 Sweden

## Abstract

*Purpose.* Examine the benefits and limitations of fine-needle aspiration cytology (FNA) used as the definitive diagnostic method before treatment.

*Method.* Review of the 25 year experience at a multidisciplinary musculo-skeletal centre where FNA is the primary diagnostic approach to soft tissue sarcoma in the extremities and trunk wall and the experience of various experts in the field.

*Results.* FNA has several benefits compared with coarse needle or open surgical biopsy. The most important are rapid preliminary diagnosis, no need for hospitalization and anaesthesia, negligible complications and fear for tumour cell spread. With the collected experience gained during the years a reliable diagnosis of sarcoma is the rule in general and specific-type diagnoses are possible in many histotypes, especially when the cytologic examination is supplemented with ancillary diagnostics. The most important limitations are inability to hit small deep-seated sarcoma and some diagnostic pitfalls such as the correct diagnosis of spindle cell neoplasms, variants of benign lipomatous tumours and ‘new soft tissue tumour entities’.

*Discussion.* Optimal use of FNA calls for certain requirements such as centralization, experience in soft tissue tumour cytology–histopathology, the FNA technique and close co-operation between the orthopaedic surgeon and cytopathologist.


*Sarcoma* (1998) **2**, 155–161

ORIGINAL ARTICLE

### Fine-needle aspiration cytology of soft tissue sarcoma: benefits and limitations

MÅNS ÅKERMAN

*Department of Pathology and Cytology, University Hospital, Lund, Sweden*

**Abstract**
*Purpose*. Examine the benefits and limitations of fine-needle aspiration cytology (FNA) used as the definitive diagnostic method before treatment.
*Method*. Review of the 25 year experience at a multidisciplinary musculo-skeletal centre where FNA is the primary diagnostic approach to soft tissue sarcoma in the extremities and trunk wall and the experience of various experts in the field.
*Results*. FNA has several benefits compared with coarse needle or open surgical biopsy. The most important are rapid preliminary diagnosis, no need for hospitalization and anaesthesia, negligible complications and fear for tumour cell spread. With the collected experience gained during the years a reliable diagnosis of sarcoma is the rule in general and specific-type diagnoses are possible in many histotypes, especially when the cytologic examination is supplemented with ancillary diagnostics. The most important limitations are inability to hit small deep-seated sarcoma and some diagnostic pitfalls such as the correct diagnosis of spindle cell neoplasms, variants of benign lipomatous tumours and 'new soft tissue tumour entities'.
*Discussion*. Optimal use of FNA calls for certain requirements such as centralization, experience in soft tissue tumour cytology–histopathology, the FNA technique and close co-operation between the orthopaedic surgeon and cytopathologist.

**Key words**: *fine needle aspiration, diagnosis, soft tissue sarcoma, benefits, limitations.*

### Introduction

Open surgical biopsy or coarse needle biopsy are widely considered to be the diagnostic procedures necessary before the definitive treatment of a soft tissue sarcoma. Fine-needle aspiration and cytodiagnosis (FNA) has not been universally accepted as a pretreatment diagnostic method although numerous reports on the use of FNA in the diagnosis of soft tissue tumours have been published during the last 20 years.[1–6] The subsequent review on the benefits and limitations of FNA as the definitive diagnostic method of soft tissue sarcoma before treatment is based on a 25 year experience of FNA of soft tissue tumours in the extremities and trunk wall from the multidisciplinary Musculo-Skeletal Tumour Centre at the University Hospital, Lund as well as on cited references.

### Benefits of FNA

FNA offers several advantages compared with open as well as coarse needle biopsy. FNA of soft tissue tumours is a harmless out-patient procedure. Severe complications are non-existent; at most there is a tenderness for some days after the needling and a local haemorrhage. Anaesthesia and hospitalization are not necessary. In children, a short general anaesthesia may be needed, depending on the clinical presentation of the tumour. When the aspirations are performed by cytopathologists it is easy to perform a rapid staining of the first smear and within 10–15 minutes ensure that the material is sufficient and diagnosable and to suggest a preliminary diagnosis. Further investigations and/or suggested treatment can then be discussed with the patient at the first visit, which is important when patients are referred from other hospitals. Properly performed FNA is the least tissue-invasive diagnostic procedure and the risk for sarcoma-cell spread is negligible.

Compared with coarse needle biopsy, which is also an out-patient procedure, a rapid preliminary report is easier to render as well as the sampling of tumour material from different parts of large tumours in order to diagnose tumour heterogeneity,

Correspondence to: M. Åkerman, Department of Pathology and Cytology, University Hospital, S-221 85 Lund, Sweden. Tel.: +46 46 173500; Fax: +46 46 143307.

1357-714X/98/030155-07 $9.00 © 1998 Carfax Publishing Ltd

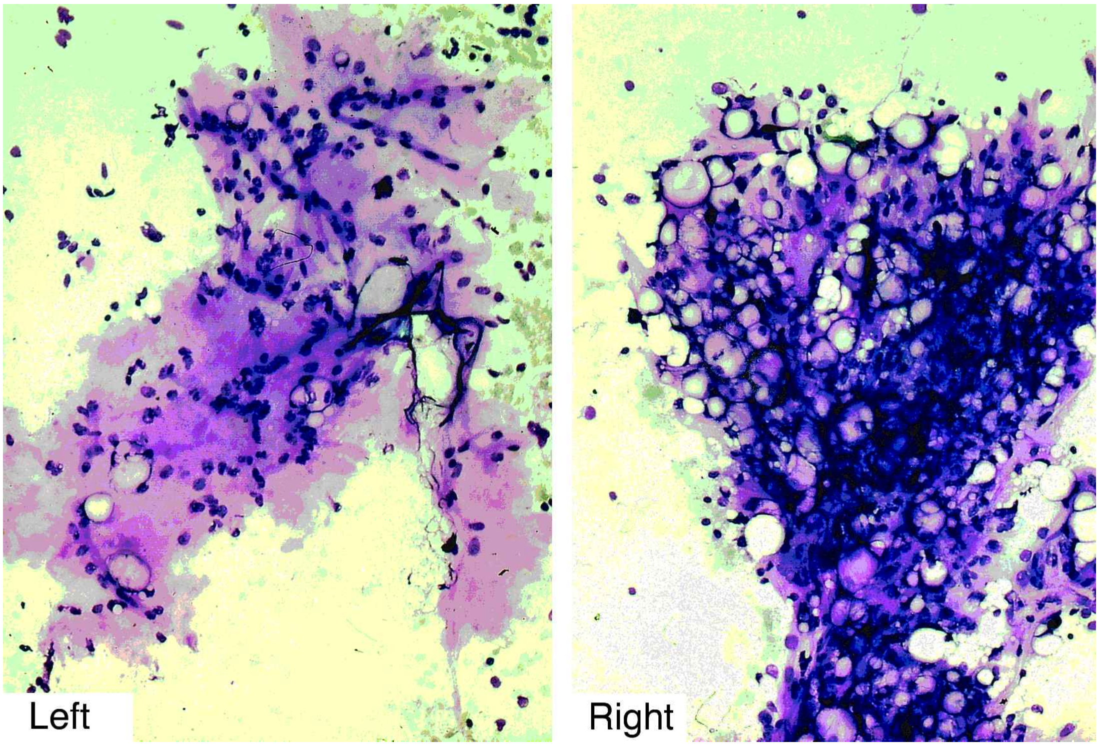

**Fig. 1.** *Part of the smears from aspirates from two different areas of a large intramuscular sarcoma. Left: typical myxoid liposarcoma with myxoid background, branching capillaries and scattered lipoblasts. Right: Cellular tumour fragment with closely packed lipoblasts corresponding to round cell liposarcoma. (MMG × 250).*

which can provide important diagnostic information (Fig. 1). The economical benefits are not only due to reduced costs for hospitalization and the use of an operating room, but the number of patient visits is also reduced.

The most important objections against the use of FNA as the definitive diagnosis of soft tissue tumours in general is the proposed inability for the cytopathologist to give a reliable histotype diagnosis and malignancy grade.[7,8] The benefits and limitations of FNA in the definitive diagnosis of a soft tissue sarcoma must, however, be related to the proposed treatment. When primary surgery is considered, the surgeon must have a reliable diagnosis of sarcoma. The type of surgery performed depends more on the site and size of the sarcoma and its relation to vessels, nerve bundles and periosteum than on the histogenetic type. Thus with this treatment modality the cytopathologist must be able to exclude other malignancies as soft tissue metastases and primary soft tissue malignant lymphoma and to differentiate between benign soft tissue tumours/lesions and sarcoma. On the other hand, when the treatment option is neoadjuvant therapy followed by surgery, the FNA diagnosis must equal that of a biopsy or coarse needle specimen with regard to specific sarcoma type and malignancy grade.

### Primary surgery of soft tissue sarcoma

At present the fine-needle aspiration cytology of numerous benign soft tissue tumours/lesions as well as soft tissue sarcoma histotypes have been investigated and diagnostic criteria suggested (Table 1).

One important group of lesions in which the benefits of FNA are well documented is in the diagnosis of the various pseudosarcomatous soft tissue lesions (nodular fasciitis, proliferative fasciitis and myositis and pseudomalignant myositis ossificans). The clinical presentation of these lesions is often suspicious for malignancy; rapid growth, firm at palpation and fixed to underlying structures and biopsy specimens or excised lesions have been misinterpreted as sarcoma. The FNA cytology of these lesions has been thoroughly studied.[9–12] It has also been documented that the need for surgical intervention most often is unnecessary as the

**Table 1.** *Soft tissue sarcomas cytologically characterized in FNA smears by correlative cytological–histological studies*

| |
|---|
| Malignant fibrous histiocytoma |
| Myxofibrosarcoma |
| Liposarcoma |
| Leiomyosarcoma |
| Synovial sarcoma |
| Clear cell sarcoma |
| Alveolar soft part sarcoma |
| Rhabdomyosarcoma |
| Ewing's sarcoma |

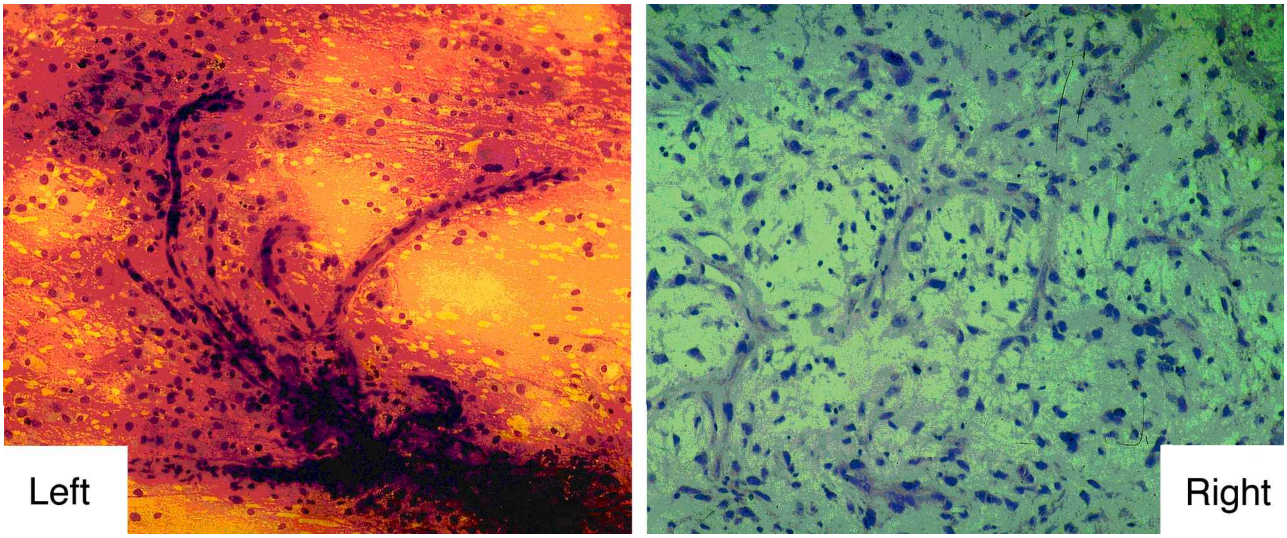

**Fig. 2.** *Low-power view of a low-grade myxofibrosarcoma. Left: aspirate; right: part of the surgical specimen. The typical histopathological features are easily observed in the smear: myxoid background matrix, curvo-linear vessels and slight atypia. (MMG and HE × 250).*

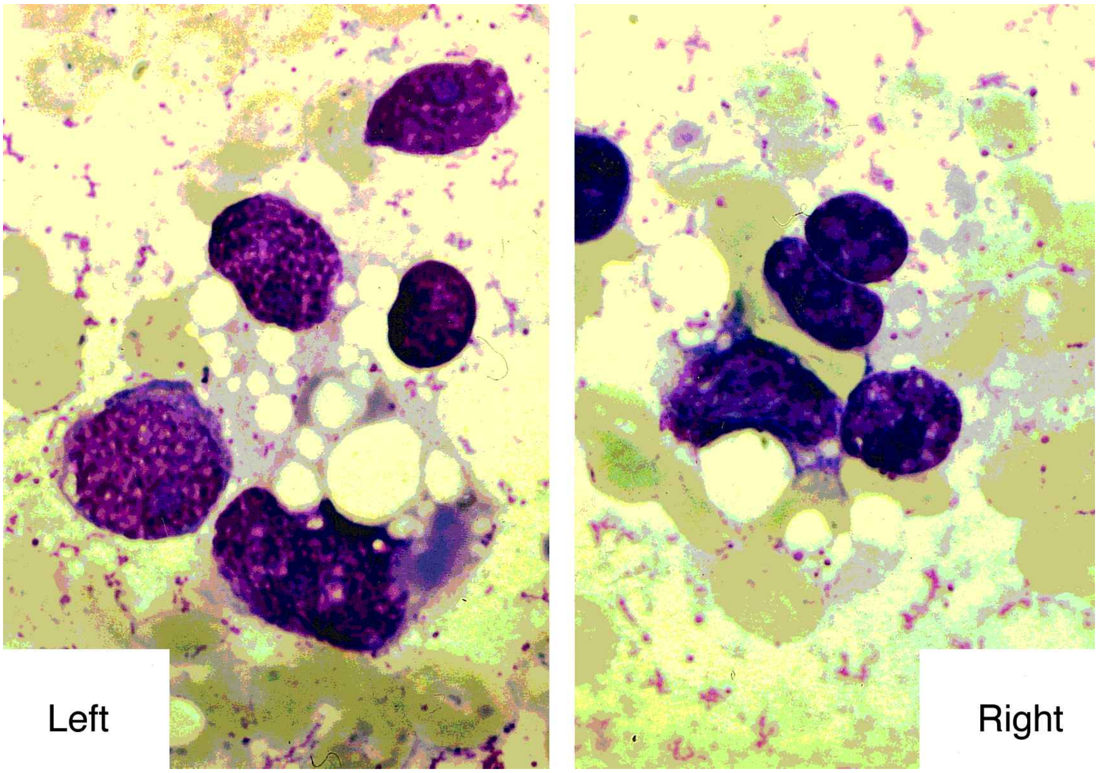

**Fig. 3.** *Highly atypical lipoblasts in an aspirate from a pleomorphic liposarcoma. (MGG × 1000).*

also been documented that the need for surgical intervention most often is unnecessary as the majority of these tumour-like lesions disappear completely or greatly diminish in size some weeks after the needling.[11,13,14]

The cytologic appearance in FNA smears of the most common sarcomas—pleomorphic sarcoma of the MFH-type, myxofibrosarcoma (Fig. 2), myxoid, round cell and pleomorphic liposarcoma (Fig. 3), leiomyosarcoma (Fig. 4) and synovial sarcoma—has been described in various series of correlative cytologic–histologic studies.[15–22] Less frequent sarcomas such as clear cell sarcoma, alveolar soft part sarcoma, rhabdomyosarcoma and (extraskeletal) Ewing's sarcoma/PNET (peripheral neuroectodermal tumour) have also been characterized in FNA material.[23–28]

There is a number of sarcoma entities which are insufficiently characterized in FNA, among them especially malignant vascular tumours. It is often possible, however, to give a confident sarcoma diagnosis in these cases, the evaluation of malignancy based on the smear pattern, the uniform or pleomorphic cytologically malignant cell population,

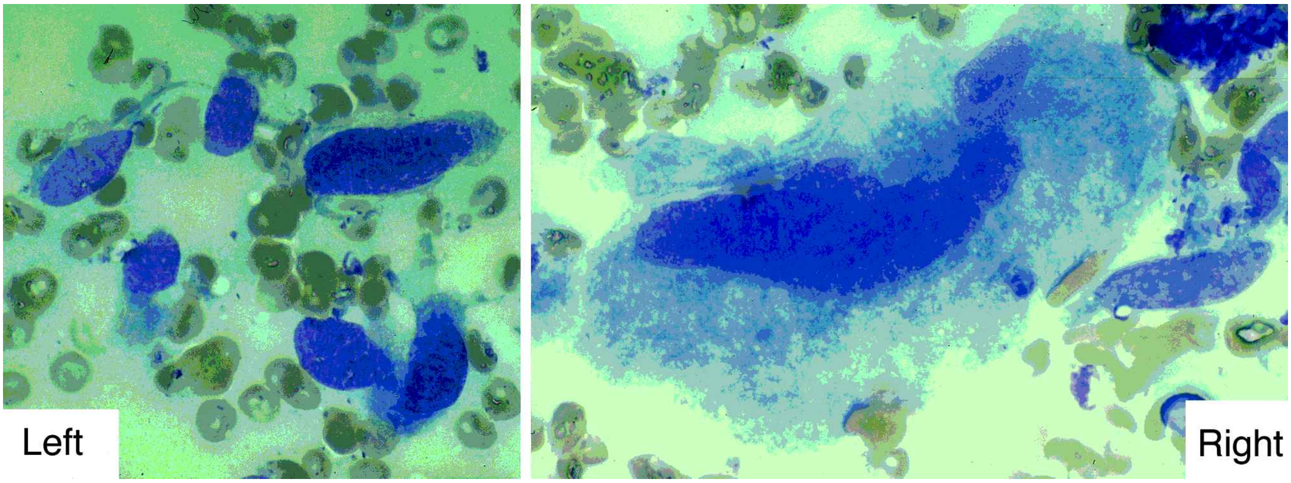

**Fig. 4.** *In pleomorphic, high-grade malignant leiomyosarcoma the typical blunt-ended cigar-shaped nuclei may be found. (MGG × 1000).*

numerous mitotic figures and fragments of necrotic tumour tissue.

### Neoadjuvant therapy followed by surgery

At present, neoadjuvant therapy is considered in the small round cell sarcomas such as rhabdomyosarcoma, extraskeletal Ewing's sarcoma, PNET, and synovial sarcoma and in selected large, deep-seated high-grade malignant soft tissue sarcoma of various histotypes.

When neoadjuvant therapy (radiotherapy or chemotherapy) is part of the definitive treatment, the cytodiagnosis must include a confident histotype diagnosis and in cases of preoperative radiotherapy of deep-seated sarcoma also a reliable malignancy grading. Cytologic malignancy grading of soft tissue sarcoma into low or high grade is possible and is based on the evaluation of cellular and nuclear pleomorphism and atypia, mitotic activity, especially the presence of atypical mitoses, and necrosis. The histogenetic type of a sarcoma might also be important in the decision of malignancy grade, for example, pure myxoid liposarcoma is most often a low-grade tumour while synovial sarcoma is high-grade. According to our experience a reliable malignancy grading into low or high grade malignant sarcoma is possible to perform in about 75% of sarcomas needled.[29]

The cytologic features of rhabdomyosarcoma, Ewing's sarcoma, PNET, and synovial sarcoma in FNA smears have been studied and diagnostic criteria defined. Although these criteria may be the basis of a confident type-diagnosis the cytologic examination should be supplemented with various ancillary diagnostic methods. In FNA aspirates it is possible to make use of the same supplementary techniques as in surgical specimens and the use of adjunctive diagnostic methods in FNA is well documented.[25,30–34]

At present routine cytologic examination combined with ancillary diagnostics successfully and reliably can diagnose most small round cell sarcomas and synovial sarcomas before the neoadjuvant therapy. In many pleomorphic high-grade sarcomas it is also possible to define the histotype.

### Limitations of FNA

The limitations of FNA in the diagnosis of soft tissue sarcoma are threefold: (a) the sarcoma is not hit with the needle and a false diagnosis may be rendered on the reactive cellular changes in the aspirated surrounding tissues; (b) insufficient material (poor yield or technically inferior smears) from the tumour is evaluated; and (c) misinterpretation of the aspirated cells. According to our 25 year experience one important reason for a false diagnosis is that the material examined does not originate from the sarcoma but from the surrounding tissues. In these cases either a benign diagnosis is given or a false sarcoma-type diagnosis is suggested, based on reactive, pseudomalignant cellular changes, especially reactive changes in adipose tissue resembling liposarcoma or reactive pleomorphic fibroblasts and myofibroblasts falsely interpreted as originating from a malignant fibrous histiocytoma. This is most often the case with small deep-seated, inter- or intramuscular sarcomas.

The main reason for a false diagnosis is, however, misinterpretation of the cellular material. There is a number of well-documented diagnostic difficulties. One of the most common is the correct evaluation of tumours predominantly composed of spindle cells[2,35,36] (Figs 5 and 6). Another important pitfall is false malignant diagnosis of benign lipoma variants as pleomorphic and spindle cell lipoma, hibernoma and lipoblastoma.[17] A rare diagnostic difficulty is the misinterpretation of aspirates from soft tissue metastases from pleomorphic carcinoma

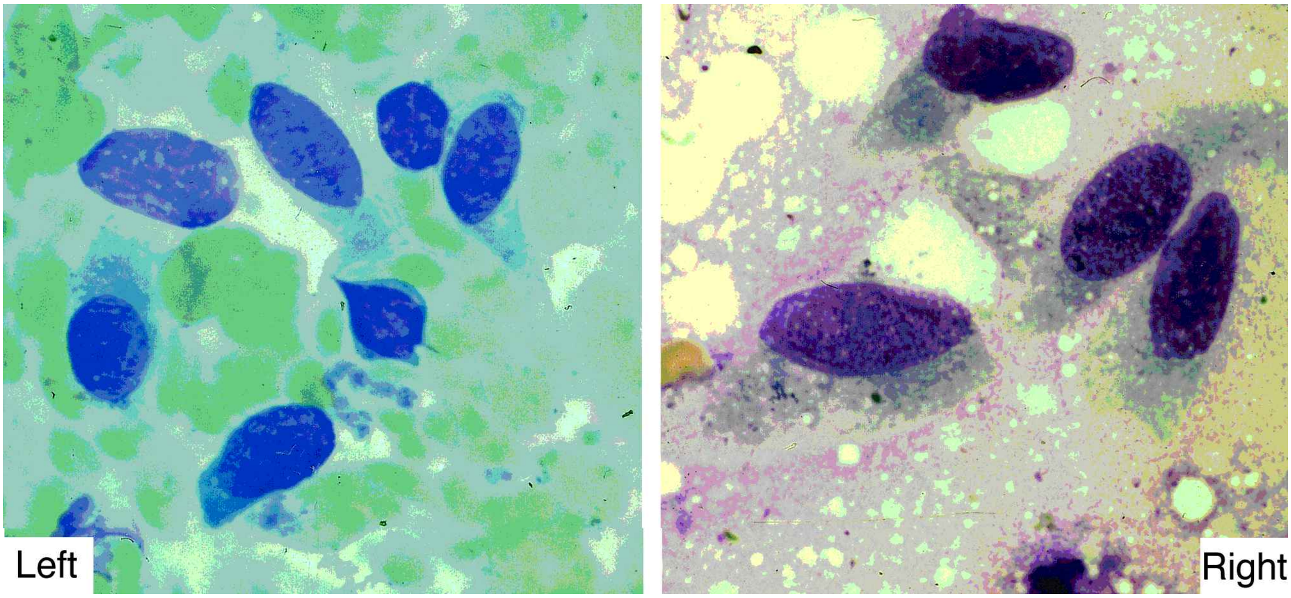

**Fig. 5.** *A poor yield from two different spindle cell neoplasms. Left: monophasic synovial sarcoma. Right: desmoid fibromatosis. Similar cellular shape and nuclear structure. A correct diagnosis is not possible.*

or melanoma or primary large cell anaplastic lymphoma as pleomorphic sarcoma and metastases from renal clear cell carcinoma as pure round cell liposarcoma.

Yet another limitation to render a correct diagnosis of a soft tissue sarcoma are 'new entities', of benign or borderline soft tissue tumours. These new entities are often rare tumours and experience of histological–cytological correlative studies of reasonably large materials are difficult to reach. Examples of new entities which may be falsely diagnosed as sarcoma are chondroid lipoma, perineurioma and solitary fibrous tumour.

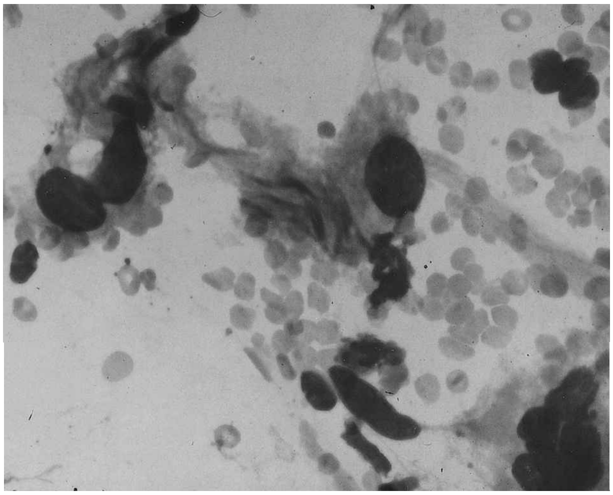

**Fig. 6.** *Part of an aspirate from an ancient neurilemoma. A low-grade malignant spindle cell sarcoma (leiomyosarcoma or malignant peripheral nerve sheet tumour) may display similar cellular features. (MGG × 1000).*

### Discussion

The clinical benefits of FNA in the primary, definitive diagnosis of soft tissue sarcoma are advantageous, but to reach a reasonably high diagnostic accuracy certain requirements should be observed. A close co-operation between the surgeon and cytopathologist is mandatory. The surgeon often will decide the insertion point of the needle and when demanding a preliminary report, discuss the diagnosis with the cytopathologist in relation to the clinical history, palpatory findings and radiographic investigations, if any. The final diagnosis should be the combined evaluation of clinical and radiographic data and the cytodiagnosis. In order to avoid false diagnoses due to a faulty aspiration technique and inability to hit the tumour at needling the cytopathologist must be a trained aspirator with a fair knowledge of the clinical behaviour and histopathology of soft tissue tumours. Ultrasound-guided aspirations of small deep-seated sarcoma are recommended to ensure that the needle is within the tumour.

As soft tissue sarcomas are relatively rare tumours (in Sweden the annual incidence is 2–3/$10^6$ inhabitants) and many benign soft tissue tumours clinically are suspected to be sarcomas, the optimal use of FNA is reached when patients with suspected sarcoma are referred with virgin tumours to multidisciplinary musculo-skeletal centres. Benign soft tissue tumours/lesions are far more common than soft tissue sarcomas, in order to ensure that the FNA of the majority of sarcomas are performed at

**Table 2.** *Recommended guidelines for referral of patients with soft tissue tumours to multidisciplinary musculo-skeletal centres*

| | |
|---|---|
| 1. | Subcutaneous tumours > 5 cm |
| 2. | All deep-seated (inter- or intramuscular) tumours |
| 3. | Soft tissue tumours, clinically suspected for malignancy |
| 4. | All soft tissue tumours in children |

multidisciplinary centres, but where most patients with benign tumours (especially lipomas) and benign reactive tumour-like lesions are not referred we have recommended some guidelines which are based on epidemiological data[37,38] (Table 2).

One challenge for the cytopathologist is the type-diagnosis of high-grade deep-seated sarcoma that are candidates for preoperative radiotherapy. When the therapy is successful it might be difficult to diagnose reliably the histogenetic type on the surgical sample. Immunocytochemistry may be helpful in suggesting smooth muscle or Schwann cell differentiation, but desmin positivity may be patchy in pleomorphic leiomyosarcoma and many malignant peripheral nerve sheet tumours do not react with the anti S-100 protein antibody. Immunocytochemistry is, however, of help in the differential diagnosis of soft tissue carcinoma metastases and soft tissue anaplastic large cell lymphoma versus pleomorphic sarcoma. Electron microscopic examination of aspirated material is a valuable diagnostic adjunct in pleomorphic leiomyosarcoma and to certify a diagnosis of round cell or pleomorphic liposarcoma. The diagnosis of monophasic synovial sarcoma is a well-known problem in FNA cytodiagnosis, but we have had the decisive help of electron microscopic studies in a number of cases.[22]

DNA-ploidy analysis is a limited but valuable adjunct. An unequivocal non-diploid cell population is compatible with malignancy and according to our experience high-grade sarcoma.[31] The small round cell sarcomas are safely diagnosed by the combined evaluation of the cytologic features and ancillary diagnostics. With the aid of immunocytochemical staining and ultrastructural investigations rhabdomyosarcoma, extraskeletal Ewing's sarcoma and PNET are confidently diagnosed. Desmin, CD99 ($MIC^2$-antigen), neuron-specific enolase and chromogranin are valuable antibodies in the type-diagnosis of these tumours. Cytogenetic and molecular genetic analyses have emerged as powerful diagnostic tools in those sarcoma where diagnostic translocations have been described and the genes involved identified. The use of cytogenetics in the diagnosis is documented in Ewing's sarcoma and synovial sarcoma[32,33] and it should be possible to make use of the known molecular genetic aberrations in the diagnosis of liposarcomas with a component of myxoid liposarcoma and alveolar rhabdomyosarcoma. We have recently used RT-PCR for the diagnosis of the PCR for the diagnosis of the *SSX1/SYT* fusion genes in a case of suspected synovial sarcoma.

The benefits of FNA in the definitive diagnosis of soft tissue sarcoma outweigh the limitations when (i) there is a close co-operation between cytopathologist and surgeon (referral of patients to multidisciplinary centres), (ii) the diagnosis is based on reliable and reproducible criteria, (iii) ancillary methods are part of the diagnosis and (iv) the diagnostic pitfalls are identified.

### References

1 Åkerman M, Idvall I, Rydholm A. Cytodiagnosis of soft tissue tumours and tumourlike conditions by means of fine-needle aspiration biopsy. *Arch Orthop Traumat* 1980; 96:61–7.
2 Åkerman M, Rydholm A, Persson BM. Aspiration cytology of soft-tissue tumours. The 10-year experience at an orthopedic oncology center. *Acta Orthop Scand* 1985; 56:407–12.
3 Miralles TG, Gosalbez F, Menendez P, Astudillo A, Torre C, Buesa J. Fine-needle aspiration cytology of soft-tissue lesions. *Acta Cytol* 1986; 30:671–8.
4 Nguyen G-K. What is the value of fine-needle aspiration biopsy in the cyto-diagnosis of soft-tissue tumours? *Diagn Cytopathol* 1988; 4:352–5.
5 Gonzales-Campora R, Munos-Arias G, Otal-Salaverri C, Jorda-Heras M, Garcia-Alvarez E, Gomez-Pascual A, Garrido-Cintado A, Heria-Vasquez A, Sanchez-Gallego F, Galera-Davidsson H. Fine needle aspiration cytology of primary soft tissue tumours. Morphologic analysis of the most frequent types. *Acta Cytol* 1992; 36:905–17.
6 Willén H, Åkerman M, Carlén B. Fine-needle aspiration (FNA) in the diagnosis of soft tissue tumours; a review of 22 years experience. *Cytopathology* 1995; 6:236–47.
7 Hajdu SI, Melamed MR. Limitations of aspiration cytology in the diagnosis of primary malignant neoplasms. *Acta Cytol* 1984; 29:337–45.
8 Hajdu SI. Diagnosis of soft tissue sarcomas on aspiration smears. *Acta Cytol* 1996; 40:607–8.
9 Dahl I, Åkerman M. Nodular fasciitis: a correlative cytological and histological study of 13 cases. *Acta Cytol* 1981; 25:215–23.
10 Reif RM. The cytologic picture of proliferative myositis. *Acta Cytol* 1982; 26:376–7.
11 Rööser B, Herrlin K, Rydholm A, Åkerman M. Pseudomalignant myositis ossificans. Clinical, radiologic, and cytologic diagnosis in 5 cases. *Acta Orthop Scand* 1989, 60(4):457–60.
12 Lundgren L, Kindblom L-G, Falkmer U, Angervall L. Proliferative myositis and fasciitis. A light and electron microscopic, cytologic, DNA cytometric and immunohistochemical study. *APMIS* 1992; 100:437–48.
13 Stanley M, Skoog L, Tani E, Horwitz C. Spontaneous resolution of nodular fasciitis following diagnosis by fine needle aspiration. *Acta Cytol* 1991; 35:616–7.
14 Willén H, Åkerman M, Rydholm A. Fine needle aspiration of nodular fasciitis—no need for surgery. *Acta Orthop Scand* 1995; 66 (Suppl. 265):54–5.

15 Walaas L, Angervall L, Hagmar B, Säve-Söderberg J. Correlative cytologic and histologic study of malignant fibrous histiocytoma: an analysis of 40 cases examined by fine-needle aspiration cytology. *Diagn Cytopathol* 1986; 2:46–54.
16 Berardo M, Powers C, Wakely P, Almeida MO, Frable W. Fine needle aspiration cytopathology of MFH. *Cancer (Cancer cytopathology)* 1997; 81:228–37.
17 Walaas L, Kindblom L-G. Lipomatous tumours. A correlative cytologic and histologic study of 27 tumours examined by fine-needle aspiration cytology. *Human Pathol* 1985; 16:6–18.
18 Åkerman M, Rydholm A. Aspiration cytology of lipomatous tumours. A 10 year experience at an orthopedic oncology center. *Diagn Cytopathol* 1987; 3:295–302.
19 Dahl I, Hagmar B, Angervall L. Leiomyosarcoma of the soft tissue. A correlative cytological and histological study of 28 cases. *Acta Pathol Microbiol Immunol Scand (A)* 1981; 89:285–91.
20 Willén H, Åkerman M, Carlén B. Fine-needle aspiration (FNA) of soft tissue leiomyosarcoma. Abstract 22, The 24th Meeting of the Scandinavian Sarcoma Group, April 22–24, 1998.
21 Merck C, Hagmar B. Myxofibrosarcoma. A correlative cytologic and histologic study of 13 cases examined by fine needle aspiration. *Acta Cytol* 1980; 24:137–44.
22 Åkerman M, Willén H, Carlén B. Fine needle aspiration (FNA) of synovial sarcoma—a comparative histological–cytological study of 15 cases, including immunohistochemical, electron microscopic and cytogenetic examination and DNA-ploidy analysis. *Cytopathology* 1996; 7:187–200.
23 Caraway N, Fanning Ch, Wojcik E, Staerkel G, Benjamin R, Ordonez N. Cytology of malignant melanoma of soft parts: fine needle aspirates and exfoliative specimens. *Diagn Cytopathol* 1993; 9:632–8.
24 Shabb N, Fanning Ch, Dekmezian R. Fine needle aspiration cytology of alveolar soft part sarcoma. *Diagn Cytopathol* 1991; 7:293–8.
25 Akhtar M, Ali M, Bakry M, Hug M, Sackey K. Fine-needle aspiration biopsy diagnosis of rhabdomyosarcoma. Cytologic, histologic and ultrastructural correlations. *Diagn Cytopathol* 1992; 8:465–74.
26 Åkerman M, Willén H, Carlén B. Fine-needle aspiration cytology of rhabdomyosarcoma. Is a reliable type diagnosis possible to render? A retrospetive study of 23 cases. *Acta Orthop Scand* 1996, 67 (suppl. 272):55.
27 Dahl I, Åkerman M, Angervall L. Ewing's sarcoma of bone. A correlative cytological and histological study of 14 cases. *Acta Pathol Microbiol Immunol Scand* 1986; 94:363–9.
28 Renshaw A, Perez-Atayade A, Fletcher J, Granter S. Cytology of typical and atypical Ewing's sarcoma/PNET. *Am J Clin Pathol* 1996; 106:620–4.
29 Åkerman M.. The cytology of soft tissue tumours. *Acta Orthop Scand* 1997; 68 (Suppl. 273):54–9.
30 Nordgren H, Åkerman M. Electron microscopy of fine needle aspiration biopsy from soft tissue tumours. *Acta Cytol* 1982; 26:179–88.
31 Kindblom L-G, Walaas L. Ultrastructural studies in the preoperative cytologic diagnosis of soft tissue tumours. *Sem Diagnostic Pathol* 1985; 3:317–44.
32 Åkerman M, Killander D, Rydholm A, Rööser B. Aspiration of musculoskeletal tumours for cytodiagnosis and DNA analysis. *Acta Orthop Scand* 1987; 58:523–28.
33 Molenaar WM, van den Berg E, Dolfin AC, Zorgdrager H, Hoekstra HJ. Cytogenetics of fine-needle aspiration biopsies of sarcoma. *Cancer Genet Cytogenet* 1995; 84:27–31.
34 Åkerman M, Dreinhöfer K, Rydholm A, Willén H, Mertens F, Mitelman F, Mandahl N. Cytogenetic studies on fine-needle aspiration samples from osteosarcoma and Ewing's sarcoma. *Diagn Cytopathol* 1996; 15:17–22.
35 Powers C, Berardo M, Frable W. Fine needle aspiration biopsy: pitfalls in the diagnosis of spindle-cell lesions. *Diagn Cytopathol* 1994; 10:232–42.
36 Ryd W, Mugal S, Ayyash K. Ancient neurilemoma, a pitfall in the cytologic diagnosis of soft tissue tumours. *Diagn Cytopathol* 1986; 2:244–7.
37 Rydholm A. Centralisation of soft tissue sarcoma. The southern Sweden experience. *Acta Orthop Scand* 1997; 68 (Suppl. 273):4–9.
38 Myhre Jensen O. A consecutive 7-year series of 1331 benign soft tissue tumours. *Acta Orthop Scand* 1981; 52:287–93.

## References

[PDF_00502] Akerman M., Dreinhöfer K., Rydholm A., Willén H., Mertens F., Mitelman F., Mandahl N. (1996). Cytogenetic studies on fine-needle aspiration samples from osteosarcoma and Ewing's sarcoma.. Diagn Cytopathol.

[PDF_00493] Akerman M., Killander D., Rydholm A., Röser B. (1987). Aspiration of musculoskeletal tumors for cytodiagnosis and DNA analysis.. Acta Orthop Scand.

[PDF_00435] Akerman M., Rydholm A. (1987). Aspiration cytology of lipomatous tumors: a 10-year experience at an orthopedic oncology center.. Diagn Cytopathol.

[PDF_00376] Akerman M., Rydholm A., Persson B. M. (1985). Aspiration cytology of soft-tissue tumors. The 10-year experience at an orthopedic oncology center.. Acta Orthop Scand.

[PDF_00467] Akhtar M., Ali M. A., Bakry M., Hug M., Sackey K. (1992). Fine-needle aspiration biopsy diagnosis of rhabdomyosarcoma: cytologic, histologic, and ultrastructural correlations.. Diagn Cytopathol.

[PDF_00427] Berardo M. D., Powers C. N., Wakely P. E., Almeida M. O., Frable W. J. (1997). Fine-needle aspiration cytopathology of malignant fibrous histiocytoma.. Cancer.

[PDF_00459] Caraway N. P., Fanning C. V., Wojcik E. M., Staerkel G. A., Benjamin R. S., Ordóez N. G. (1993). Cytology of malignant melanoma of soft parts: fine-needle aspirates and exfoliative specimens.. Diagn Cytopathol.

[PDF_00476] Dahl I., Akerman M., Angervall L. (1986). Ewing's sarcoma of bone. A correlative cytological and histological study of 14 cases.. Acta Pathol Microbiol Immunol Scand A.

[PDF_00402] Dahl I., Akerman M. (1981). Nodular fasciitis a correlative cytologic and histologic study of 13 cases.. Acta Cytol.

[PDF_00439] Dahl I., Hagmar B., Angervall L. (1981). Leiomyosarcoma of the soft tissue. A correlative cytological and histological study of 11 cases.. Acta Pathol Microbiol Scand A.

[PDF_00386] González-Cámpora R., Muñoz-Arias G., Otal-Salaverri C., Jorda-Heras M., García-Alvarez E., Gomez-Pascual A., Garrido-Cintado A., Hevia-Vázquez A., Sánchez-Gallego F., Galera-Davidson H. (1992). Fine needle aspiration cytology of primary soft tissue tumors. Morphologic analysis of the most frequent types.. Acta Cytol.

[PDF_00400] Hajdu S. I. (1996). Diagnosis of soft tissue sarcomas on aspiration smears.. Acta Cytol.

[PDF_00397] Hajdu S. I., Melamed M. R. (1984). Limitations of aspiration cytology in the diagnosis of primary neoplasms.. Acta Cytol.

[PDF_00490] Kindblom L. G., Walaas L., Widéhn S. (1986). Ultrastructural studies in the preoperative cytologic diagnosis of soft tissue tumors.. Semin Diagn Pathol.

[PDF_00411] Lundgren L., Kindblom L. G., Willems J., Falkmer U., Angervall L. (1992). Proliferative myositis and fasciitis. A light and electron microscopic, cytologic, DNA-cytometric and immunohistochemical study.. APMIS.

[PDF_00449] Merck C., Hagmar B. (1980). Myxofibrosarcoma: a correlative cytologic and histologic study of 13 cases examined by fine needle aspiration cytology.. Acta Cytol.

[PDF_00380] Miralles T. G., Gosalbez F., Menéndez P., Astudillo A., Torre C., Buesa J. (1986). Fine needle aspiration cytology of soft-tissue lesions.. Acta Cytol.

[PDF_00497] Molenaar W. M., van den Berg E., Dolfin A. C., Zorgdrager H., Hoekstra H. J. (1995). Cytogenetics of fine needle aspiration biopsies of sarcomas.. Cancer Genet Cytogenet.

[PDF_00515] Myhre-Jensen O. (1981). A consecutive 7-year series of 1331 benign soft tissue tumours. Clinicopathologic data. Comparison with sarcomas.. Acta Orthop Scand.

[PDF_00383] Nguyen G. K. (1988). What is the value of fine-needle aspiration biopsy in the cytodiagnosis of soft-tissue tumors?. Diagn Cytopathol.

[PDF_00506] Powers C. N., Berardo M. D., Frable W. J. (1994). Fine-needle aspiration biopsy: pitfalls in the diagnosis of spindle-cell lesions.. Diagn Cytopathol.

[PDF_00405] Reif R. (1982). The cytologic picture of proliferative myositis.. Acta Cytol.

[PDF_00480] Renshaw A. A., Perez-Atayde A. R., Fletcher J. A., Granter S. R. (1996). Cytology of typical and atypical Ewing's sarcoma/PNET.. Am J Clin Pathol.

[PDF_00509] Ryd W., Mugal S., Ayyash K. (1986). Ancient neurilemmoma: a pitfall in the cytologic diagnosis of soft-tissue tumors.. Diagn Cytopathol.

[PDF_00512] Rydholm A. (1997). Centralization of soft tissue sarcoma. The southern Sweden experience.. Acta Orthop Scand Suppl.

[PDF_00407] Röser B., Herrlin K., Rydholm A., Akerman M. (1989). Pseudomalignant myositis ossificans. Clinical, radiologic, and cytologic diagnosis in 5 cases.. Acta Orthop Scand.

[PDF_00464] Shabb N., Sneige N., Fanning C. V., Dekmezian R. (1991). Fine-needle aspiration cytology of alveolar soft-part sarcoma.. Diagn Cytopathol.

[PDF_00422] Walaas L., Angervall L., Hagmar B., Säve-Söderbergh J. (1986). A correlative cytologic and histologic study of malignant fibrous histiocytoma: an analysis of 40 cases examined by fine-needle aspiration cytology.. Diagn Cytopathol.

[PDF_00431] Walaas L., Kindblom L. G. (1985). Lipomatous tumors: a correlative cytologic and histologic study of 27 tumors examined by fine needle aspiration cytology.. Hum Pathol.

